# QiShenYiQi Pills, a Compound Chinese Medicine, Ameliorates Doxorubicin-Induced Myocardial Structure Damage and Cardiac Dysfunction in Rats

**DOI:** 10.1155/2013/480597

**Published:** 2013-02-26

**Authors:** Dong-Xin Tang, Hai-Ping Zhao, Chun-Shui Pan, Yu-Ying Liu, Xiao-Hong Wei, Xiao-Yuan Yang, Yuan-Yuan Chen, Jing-Yu Fan, Chuan-She Wang, Jing-Yan Han, Ping-Ping Li

**Affiliations:** ^1^Key Laboratory of Carcinogenesis and Translational Research (Ministry of Education), Department of Integrated Traditional Chinese and Western Medicine, Peking University Cancer Hospital & Institute, 52 Fucheng Road, Beijing 100142, China; ^2^Tasly Microcirculation Research Center, Peking University Health Science Center, Beijing 100191, China; ^3^Department of Oncology, The First Affiliated Hospital of Guiyang College of TCM, Guiyang, Guizhou 550002, China; ^4^Department of Integration of Chinese and Western Medicine, School of Basic Medical Sciences, Peking University, 38 Xueyuan Road, Beijing 100191, China

## Abstract

QiShenYiQi Pills (QSYQ) is a compound Chinese medicine used for treatment of cardiovascular diseases. The present study investigated the effects of QSYQ on the Doxorubicin- (DOX-) induced disorders in rat cardiac structure and function and the possible mechanism underlying. A total of 24 male Sprague-Dawley rats were administrated by intraperitoneal injections with DOX at a dose of 2.5 mg/kg, once every day for a total of 6 times. After the 6th injection, the rats were evaluated by echocardiographic analysis, and the animals with injured heart (*n* = 14) were divided into 2 groups and further treated with (*n* = 7) or without (*n* = 7) QSYQ by gavage at a dose of 0.2 g/day, once a day, over the next 2 weeks. Two weeks after QSYQ treatment, the following variables were assessed: myocardial blood flow (MBF) by Laser-Doppler Perfusion Imager, the ratio of heart weight to body weight (HW/BW), myocardial histology, myocardial content of ATP, AMP, free fatty acids (FFAs) and AMP/ATP by ELISA, and expression of PPAR**α**, PGC-1**α**, and ATP 5D by Western blot. Statistical analysis was performed using one-way ANOVA followed by Turkey test for multiple comparisons. DOX challenge significantly increased left ventricular internal diameter and HW/BW and decreased the thickness of the left ventricular posterior wall, the left ventricle ejection fraction, and the left ventricle fractional shortening. DOX also increased AMP, FFA, and AMP/ATP, decreased ATP, and downregulated the protein content of ATP 5D, PPAR **α**, and PGC-1 **α**. All these DOX-induced cardiac insults were attenuated significantly by QSYQ treatment. These results show the potential of QSYQ to ameliorate DOX-induced disorders in cardiac structure and function; this effect may be related to the increase in myocardial ATP content via the upregulation of ATP 5D, PPAR **α**, and PGC-1 **α** and the oxidation of FFA.

## 1. Introduction

Cancer and cardiovascular diseases (CVDs) have become the main cause of death for the adults in China [1]. More importantly, the therapy of cancer may evoke or aggravate CVD due to the toxicity of antineoplastic [2]. Therefore, the development of management to limit the adverse effect of antineoplastic on cardiovascular system is of clinical significance for the therapy of cancer. 

Doxorubicin (DOX), an anthracycline antibiotic [3], is one of the most effective antineoplastic medicines at present. However, continuous use of DOX may lead to chronic congestive heart failure in a dose dependent manner, which limits its clinic application [4]. Increasing study has been published with respect to the mechanism responsible for the cardiac toxicity of DOX. The myocardial damage caused by DOX is reported to be relevant to free radicals [5], iron ion unbalanced metabolism [6], calcium overload [7], mitochondria damage [8], and cell apoptosis [9]. Recent researches revealed that DOX could also reduce myocardial ATP content [10], cause the downregulation of peroxisome proliferator-activated receptor *α* (PPAR *α*) in kidney [11]. These results suggest that metabolism disorder may be implicated in DOX-induced cardiac injury.

The activation of PPAR *α* is known to mediate the expression of cardiac fatty acid oxidation (FAO) enzyme gene, while peroxisome proliferator-activated receptor-*γ* coactivator-1 *α* (PGC-1 *α*) is the essential regulator for cardiac metabolism and function. Coactivation of PGC-1 *α* and PPAR *α* can regulate FAO [12], which plays an important role in cardiac energy metabolism and injury. Shortage of ATP is known to play a dual role in the pathogenesis of ischemia-reperfusion (I/R) injury; in addition to trigger reactive oxygen species (ROS) production, it leads to the degradation of F-actin located on thin filament and thus the abnormality of cardiac structure and function. ATP synthase *δ* (encoded by ATP 5D), as one of the subunits of ATP synthase, is critical for ATP synthesis [13]. However, the role of ATP 5D in DOX-induced cardiac injury is yet unclear. Furthermore, to date no Chinese medicine has been proven to improve cardiac structure and function via myocardial energy metabolism.

QSYQ is a compound Chinese medicine composing of Radix Astragali (RA), Salvia miltiorrhiza (SM), Panax notoginseng(PN) and rosewood. In 2003, QSYQ was approved for treating coronary heart disease and angina by the Chinese State Food and Drug Administration. Our laboratory has proved that QSYQ ameliorates pressure overload-induced cardiac hypertrophy, myocardial fibrosis, myocardial blood flow, and cardiac function [14]. Zhang et al. confirmed that QSYQ can protect against cardiac injury and fibrosis in ischemia-reperfusion rat and increase the expression of vascular endothelial growth factor (VEGF) [15]. However, it is not clear whether QSYQ can reduce DOX-induced cardiac disorder in structure and function, and, if yes, what is the underlying mechanism? The present study was conducted to address the effect of QSYQ on DOX-induced disorder by testing ATP, ATP 5D, F-actin, PPAR *α*, and PGC-1 *α* in rat heart.

## 2. Materials and Methods

### 2.1. Animals

Male Sprague-Dawley (SD) rats weighing 189 ± 12 g were purchased from the Animal Center of Peking University Health Science Center (Beijing, certificate no. SCXK 2006-0008). The animals were housed in cages at 22°C ± 2°C and humidity of 40%  ± 5% under a 12-hour light/dark cycle, received standard diet and water ad libitum. The animals were fasted for 12 h before the experiment, while allowing free access to water. The experimental procedures were carried out in accordance with the European commission guidelines (2010/63/EU). All animals were handled according to the guidelines of the Peking University Animal Research Committee. The protocols were approved by the Committee on the Ethics of Animal Experiments of the Health Science Center of Peking University (LA2011-67).

### 2.2. Reagents

QiShenYiQi Pills (QSYQ, lot number: 110708) was obtained from Tasly Pharmaceutical Co., Ltd. (Tasly, Tianjin, China), 0.5 g per pouch. Doxorubicin Hydrochloride for Injection (DOX, lot number: 9QL0161) was purchased from Pfizer Pharmaceuticals Ltd. (Pfizer, Latina, Italy), one bottle containing 10 mg of Doxorubicin Hydrochloride, 50 mg of lactose, and 1 mg of methylparaben. Isoflurane was purchased from Jiupai Pharmaceutical Co. Ltd. of HeBei (Jiupai, Shijiazhuang, China). Tetramethylethylenediamine and ammonium persulfate were purchased from Sigma-Aldrich Co. (Sigma, St. Louis, MO, USA). Supper ECL Plus was purchased from Pierce Biotechnology, Inc. (Pierce, Rockford, IL, USA). RIPA tissue lysate was purchased from Cell Signaling Technology, Inc. (CST, Danvers, MA, USA). Protease inhibitor was obtained from Merck Drugs & Biotechnology Co. (Merck, Darmstadt, Germany). Protein marker was purchased from MBI Fermentas (MBI, Burlington, ON, Canada). Whole protein extracting kit was purchased from Bio-Rad Laboratories, Inc. (Bio-Rad, Hercules, CA, USA). BCA protein assay kit was purchased from Bio-Rad Laboratories, Inc. (Bio-Rad, Hercules, CA, USA). ELISA kit for ATP, AMP, and free fatty acid (FFA) of rat was purchased from R & D Systems, Inc. (R & D, Minneapolis, USA). The antibodies against PPAR *α* and PGC-1 *α* were purchased from Abcam, Inc. (Abcam, Cambridge, USA). The antibody against ATP 5D was purchased from Santa Cruz Biotechnology (Santa Cruz, CA, USA). All other reagents were of reagent grade.

### 2.3. Animal Model and Drug Administration

Rats were randomly divided into 5 groups: Control 2W (*n* = 6), Control 4W (*n* = 6), DOX 2W (*n* = 14), DOX 4W + NS (*n* = 7), and DOX 4W + QSYQ group (*n* = 7). For the last three groups, twenty-four rats were administrated with DOX (2.5 mg/kg in saline) by intraperitoneal injection once every other day for a total of 6 times, as described previously by others [16]. After the sixth injection, left ventricular ejection fraction (LVEF) was measured by echocardiographic analysis. Of 24 rats tested, fourteen had LVEF reduced by 10%, which were scored as DOX-injured animals (DOX 2W) and used for subsequent experiments. The DOX-injured rats were further randomly divided into DOX 4W + NS group and DOX 4W + QSYQ group. The animals in the DOX 4W + NS group were administrated with 1 mL of saline daily by gavage for the subsequent 2 weeks. Over the same period of time, animals in the DOX 4W + QSYQ group received 1 mL of QSYQ saline solution (concentration of 0.2 g/mL), as described [14], instead of saline alone, daily by gavage. The rats in the Control 2W group were administrated with saline by intraperitoneal injection at the dose of 1 mL every other day for 14 days, while the animals in Control 4W group were treated in the same way as those in Control 2W group, and followed by administration of saline once a day by gavage for subsequent 2 weeks.

### 2.4. Echocardiographic Analysis

The left ventricular wall thickness and cardiac function were evaluated at week 2 and 4, respectively, using a Vevo 770 High-Resolution Imaging System (Vevo 770, Visual Sonics Inc, Toronto, ON, Canada) with a 17.5 MHz linear array transducer (model 716). The following parameters were measured as indicators of cardiac function and remodeling: left ventricular internal diameter at end-diastole (LVIDd), left ventricular internal diameters at end-systole (LVIDs), left ventricular posterior wall at diastole (LVPWd), left ventricular posterior wall at systole (LVPWs), left ventricle ejection fraction (%EF), and left ventricle fractional shortening (%FS) [14].

### 2.5. Measurement of Myocardial Blood Flow (MBF)

Images of MBF in the territory supplied by left anterior descending coronary artery were acquired by Laser-Doppler Perfusion Imager (PeriScan PIM3, Perimed, Stockholm, Sweden) equipped with a computer and evaluated on an area of 3 × 4 mm^2^ with the software LDPI win 3.1 (LDPIwin 3.1, Perimed, Stockholm, Sweden) [17].

### 2.6. Measurement of HW/BW

Rats were killed at the end of experiment, and the hearts were removed and washed with normal saline. Both body weight (BW) and heart weight (HW) were determined by an electronic balance (CPA64-0CE, Sartorius AG, Goettingen, Germany), and the ratio of HW to BW (HW/BW) was calculated [14].

### 2.7. Histological Investigation of Myocardial Tissues

Hearts were removed at the end of the experiment, fixed in 4% formaldehyde, and further prepared for paraffin sectioning. The paraffin sections (5 *μ*m) were rehydrated and stained with hematoxylin and eosin (HE). The images were captured by a digital camera connected to a stereo microscope (SZ-40, Olympus, Tokyo, Japan) and an optical microscope (Digital Sight DS-5 M-U1, Nikon, Tokyo, Japan) and processed with the software Image-Pro Plus 5.0 (Image-Pro Plus 5.0, Media Cybemetrics, Rockville, MO, USA).

### 2.8. F-Actin Staining

Paraffin sections were treated with 0.01 M sodium citrate for antigen retrieval. After washing, sections were then incubated with rhodamine phalloidin (1 : 40, R415, Invitrogen, Carlsbad, CA, USA) for 1 h at 37°C and washed with PBS. To label nucleus, the sections were incubated with Hoechst 33342 (1 : 100, Molecular Probes, New York, NY, USA) [18]. Sections were observed with a laser scanning confocal microscope (TCS SP5, Leica, Mannheim, Germany).

### 2.9. Assessment of ATP, AMP, FFA, and AMP/ATP

At the end of the experiment, the rat was perfused with NS, and a tissue block about 2 mm^3^ was removed from the heart for the assessment of the concentrations of ATP, AMP, and FFA in myocardial tissues by ELISA kits according to the manufacturer's protocol. OD values were determined by enzyme microplate reader (Thermo multiskan Mk3, Thermo Fisher Scientific Inc., Barrington, IL, USA), with a detection wave length of 450 nm. The concentrations of ATP, AMP, FFA, and AMP/ATP were calculated based on the standard curves.

### 2.10. Western Blotting Assay

A piece of about 200 mg of tissue was cut from the heart of each animal and preserved at −80°C (*n* = 3). The whole protein was extracted. The concentration of whole protein was detected in duplicate with BCA protein assay kit according to instruction, and the mean values were computed. All the concentrations of whole proteins were adjusted to the lowest concentration detected and the samples were preserved at −80°C.

The whole protein was separated on 10% SDS-PAGE and transferred to polyvinylidene difluoride membrane. The membrane was blocked with 5% nonfat dry milk or 3% BSA and, after washing, incubated overnight at 4°C with primary antibody against PPAR*α* (1 : 1000), PGC-1 *α* (1 : 500), and ATP5D (1 : 200). Following rinsing, the membranes were incubated for 1 h at room temperature with respective HRP-conjugated secondary antibody. The membranes were developed with ECL, exposed in dark box, and the protein signal was quantified by scanning densitometry in the X-film by bioimage analysis system (Image-Pro plus 6.0, Media Cybernetics, Bethesda, MD USA). The result of each group was expressed as relative optical density compared with that from Control group. 

### 2.11. Statistical Analysis

All parameters were expressed as mean ± S.E. Statistical analysis was performed using one-way ANOVA followed by Turkey test for multiple comparisons. A probability of less than 0.05 was considered to be statistically significant.

## 3. Results

### 3.1. QSYQ Ameliorates DOX-Induced Disorder in Left Ventricular Wall Thickness and Heart Function in Rats

**Figure 1:**
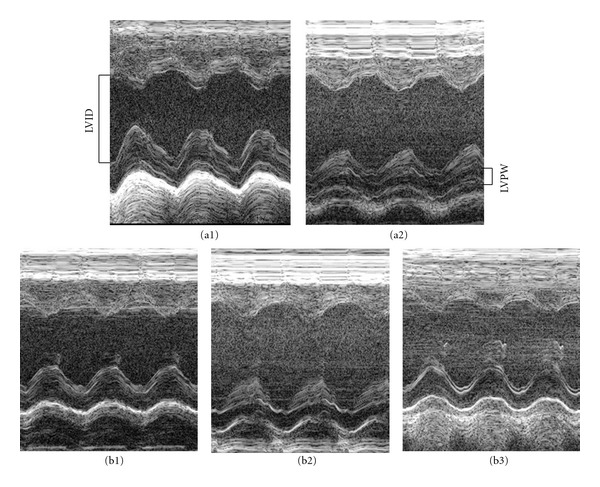
Representative rat echocardiograms in various conditions. (a1) Control 2W group, (a2) DOX 2W group, (b1) Control 4W group, (b2) DOX 4W + NS group, and (b3) DOX 4W + QYSQ group. LVID: left ventricular internal diameter at end diastole; LVPW: left ventricular posterior wall at diastole.

The results of echocardiography analysis in various groups are displayed in Table 1. Compared with Control 2W group, the DOX 2W group had a significant increase in LVIDs and a decrease in LVPWd, LVPWs, EF%, and FS%, all of which were attenuated by QSYQ treatment for 2 weeks. The representative echocardiograms in different groups are presented in Figure 1.

### 3.2. Effect of QSYQ on DOX-Induced Reduction in MBF in Rats

**Figure 2:**
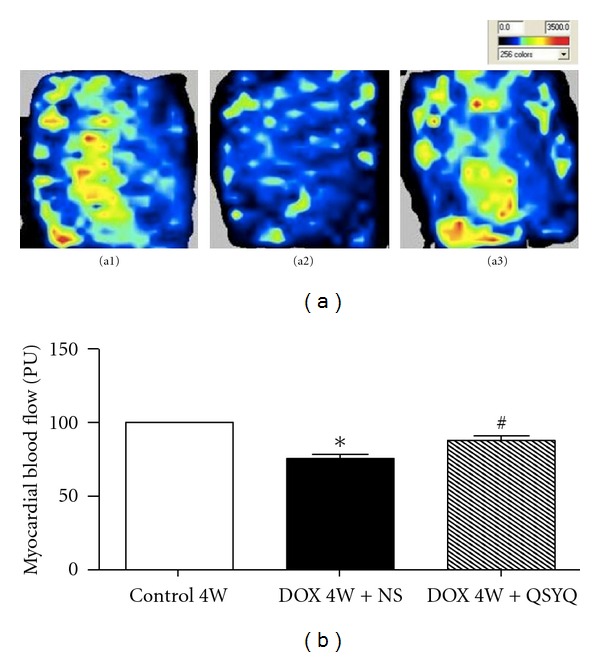
The effect of QSYQ on rat MBF. (a) the MBF images acquired by Laser-Doppler Perfusion Imager in Control 4W group ((a1), *n* = 6), DOX 4W + NS group ((a2), *n* = 7), and DOX 4W + QSYQ group ((a3), *n* = 7). Red color represents high MBF and blue color represents low MBF. (b) The statistical results of MBF in different groups. The data are presented as mean ± S.E. *P < 0.05 versus Control 4W group; ^#^ *P* < 0.05 versus DOX 4W + NS group.

Figure 2(a) shows the MBF images acquired by Laser-Doppler Perfusion Imager in different groups. Of notice, as compared to the Control 4W group, MBF apparently decreased in DOX 4W + NS group. In contrast, the image of DOX 4W + QSYQ group shows that QSYQ treatment for two weeks obviously attenuated DOX-induced decrease in MBF. This result was verified by the quantitative evaluation of myocardial coronary blood flow (Figure 2(b)).

### 3.3. Effect of QSYQ on DOX-Induced Reduction in HW/BW in Rats

**Figure 3:**
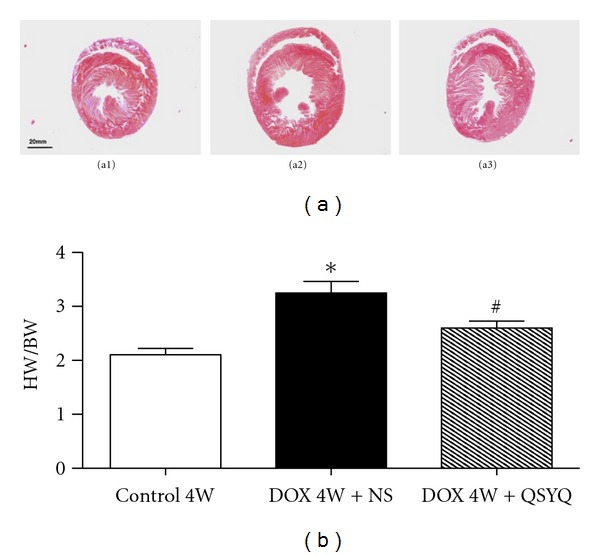
The effect of QSYQ on rat HW/BW. (a) Representative transverse heart slice from Control 4W group (a1), DOX 4W + NS group (a2), and DOX 4W + QSYQ group (a3). Myocardial tissues of rat were sampled in cross-sections 5 mm in thickness throughout the left and right ventricles. Sections were fixed in formalin and embedded in paraffin. Transverse sections were cut at 5 *μ*m thickness. (b) The statistical results of HW/BW in Control 4W group (*n* = 6), DOX 4W + NS group (*n* = 7), and DOX 4W + QSYQ group (*n* = 7). The data are presented as mean ± S.E. *P < 0.05 versus Control 4W group; ^#^ *P* < 0.05 versus DOX 4W + NS group.

Figure 3 shows the statistical results of the ratio of the HW/BW in different groups. As noticed, DOX 4W + NS group had a 30% increase in HW/BW compared to that of Control 4W group, indicating a significant myocardial hypertrophy after DOX challenge, which was significantly attenuated by 2 weeks of QSYQ treatment.

### 3.4. Effect of QSYQ on DOX-Induced Myocardial Injury in Rats

**Figure 4:**
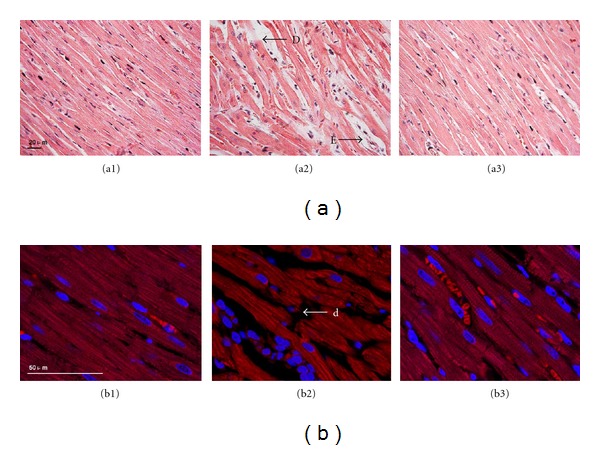
Effect of QSYQ on the structure of rat myocardial tissue. (a) Representative histological images by H & E staining in Control 4W group (a1), DOX 4W + NS group (a2), and DOX 4W + QSYQ group (a3). (b) Representative photographs of F-actin stained by rhodamine phalloidin in Control 4W group (b1), DOX 4W + NS group (b2), and DOX 4W + QSYQ group (b3). E: edema; D: disrupted myocardial fiber; d: disrupted myocardial fiber.

Figures 4(a) and 4(b) illustrate the results of histological examination of myocardial tissues in different groups. Compared with the Control 4W group (a1), distinct alterations occurred in myocardial tissues from the DOX 4W + NS group, including myocardial edema and fiber breakage (a2), all of which were noticeably ameliorated by 2-week QSYQ treatment (a3).  Figure 4(b) shows the images of rhodamine phalloidin-labeled F-actin, wherein decreased F-actin and myocardium rupture were observed in the DOX 4W + NS group (b2) in comparison to Control 4W group (b1). Administration of QSYQ for 2 weeks significantly attenuated F-actin reduction and myocardium rupture (b3).

### 3.5. Effect of QSYQ on DOX-Induced Changes in the Energy Metabolism

**Figure 5:**
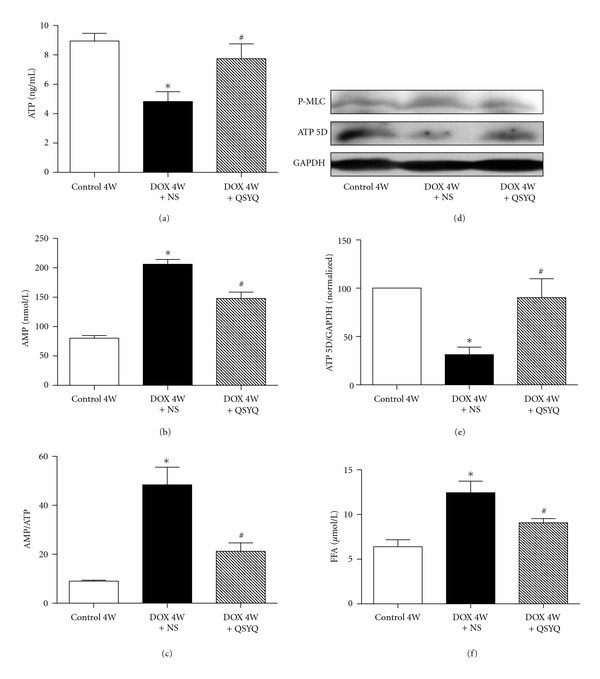
Effect of QSYQ on the content of ATP, AMP, AMP/ATP, and FFA in rat myocardial tissue. (a), (b), (c), and (f) show the statistical results from ELISA for ATP, AMP, AMP/ATP, and FFA, respectively, in Control 4W group (*n* = 6), DOX 4W + NS group (*n* = 7), and DOX 4W + QSYQ group (*n* = 7). (d) Representative Western blotting for ATP 5D in rat myocardial tissue in various groups. (e) Quantitative results of the Western blotting for ATP 5D. The data are presented as mean ± S.E. *P < 0.05 versus Control 4W group; ^#^ *P* < 0.05 versus DOX 4W + NS group.

The ATP, AMP, FFA content, and the ratio of AMP/ATP were determined by ELISA at the end of the experiment in different groups (Figure 5). In comparison with Control 4W group rats, the ATP content of rat myocardial tissue in DOX 4W + NS group significantly decreased (a), while the content of AMP (b), the ratio of AMP/ATP (c), and FFA (f) significantly increased. In DOX 4W + QSYQ group the ATP content were remarkably recovered, and so did the content of AMP, FFA, and the ratio of AMP/ATP. 

To explore the cause of the observed change in ATP content, the expression of ATP 5D was detected by Western blots. As shown in Figure 5(d), the expression of ATP 5D was reduced significantly by DOX, as compared to Control 4W group. Administration of QSYQ for 2 weeks relieved the decline of ATP 5D expression evoked by DOX. 

### 3.6. Effect of QSYQ on DOX-Induced Alteration in the Expression Level of PPAR *α* and PGC-1 *α*

**Figure 6:**
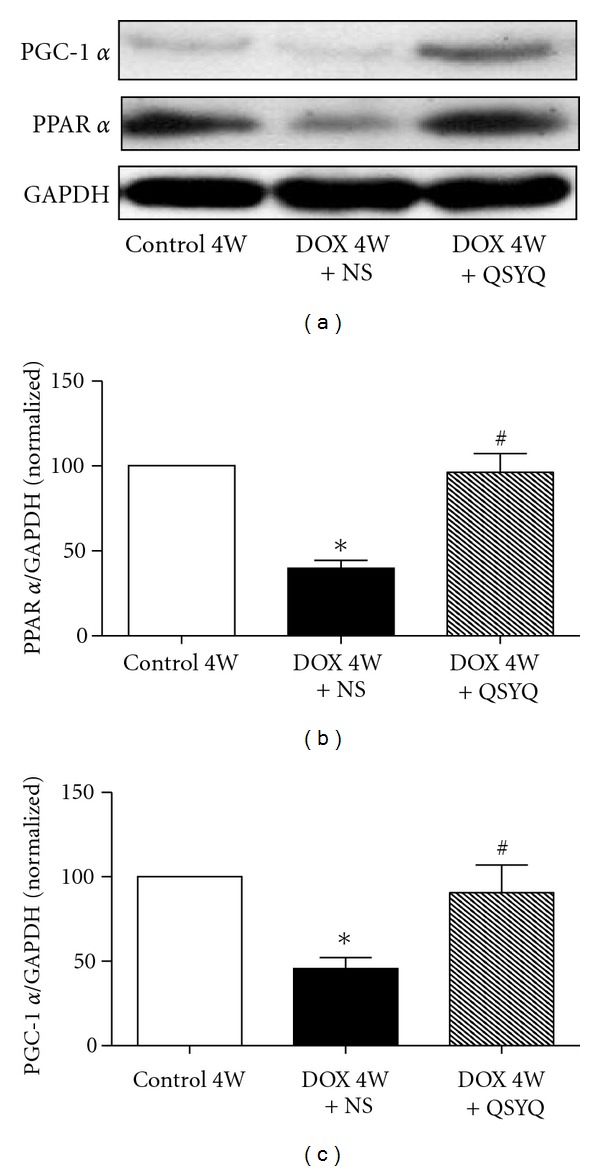
Assessment of PGC-1 *α* and PPAR *α* protein in rat myocardial structure. (a) Representative Western blotting for PGC-1 *α* and PPAR *α* in Control 4W group (*n* = 6), DOX 4W + NS group (*n* = 7), and DOX 4W + QSYQ (*n* = 7). (b) and (c) show the quantitative results of the Western blotting of PGC-1 *α* and PPAR *α*, respectively. The data are presented as mean ± S.E. *P < 0.05 versus Control 4W group; ^#^ *P* < 0.05 versus DOX 4W + NS group.

Western Blotting was undertaken to assess the expression levels of PPAR *α* and PGC-1 *α* in myocardial tissues from different groups at the end of the experiment (Figure 6). As noticed, both qualitative survey and quantitative evaluation indicate that the expression levels of PPAR *α* and PGC-1 *α* in DOX 4W + NS group were apparently decreased compared with Control 4W, while these decreases restored significantly in the DOX 4W + QSYQ group.

## 4. Discussion 

The present study revealed that the intraperitoneal injection of DOX for 2 weeks in rats leads to an increase in LVID and HW/BW, a reduction of LVPW and LVPW, and a decrease in EF and FS. In addition, DOX caused reduction of ATP synthase subunit ATP 5D, the degradation of myocardial F-actin, and myocardium rupture. All the DOX-induced alterations can be evidently ameliorated by the administration of QSYQ for 2 weeks, suggesting the potential of QSYQ to relieve the DOX-induced cardiac insufficiency. In addition, we also found that QSYQ promotes the expression of PPAR *α* and PGC-1 *α* in the DOX-injured myocardium cells, facilitates the oxidation of fatty acid, degrades myocardial free fatty acid, and finally increases the content of ATP.

Previous studies reported that intraperitoneal injection of DOX in rats provokes cardiomyopathy, exhibiting as a larger LVIDS and a thinner LVPW [19]. In the present study, using the same animal model with decreased cardiac function induced by DOX, we proved that continuous post-intervention by QSYQ for 2 weeks obviously rescues the cardiac function. Parallel with functional assessment, the beneficial role of QSYQ in DOX-induced myocardial injury was demonstrated by morphological study as well. QSYQ is a compound traditional Chinese medicine which is mainly used to promote blood circulation. Previous studies showed that QSYQ could attenuate the myocardial injury and fibrosis induced by overload pressure [14] and ischemia reperfusion [17], and improve myocardial blood flow and cardiac function in rats [14]. The present study provided evidence supporting for the effectiveness of QSYQ in ameliorating cardiac disorders in an unexplored condition, the DOX-induced myocardial injury and cardiac dysfunction. Cardiac toxicity is the main problem limiting the clinical use of DOX; the finding of the present study may open a potential avenue to surmount the adverse effect of DOX for its clinical application.

Fatty acid beta-oxidation takes place in mitochondrial matrix, a process that constitutes the major source of energy for myocardial cell activity [20]. Numerous genes are involved in fatty acid beta-oxidation, which are primarily regulated byPPAR *α*/PGC-1 *α* complex [12], with PGC-1 playing an important role in regulating cardiac mitochondrial number and function [21]. In line with others, the present study revealed an upregulating cardiac FFA level and downregulating myocardial ATP content in rats after DOX challenge [22]. Importantly, we demonstrated for the first time that administration of QSYQ to the DOX-challenged rats for 2 weeks remarkably suppressed myocardial FFA level and elevated cardiac ATP content, meanwhile, increased PPAR *α*/PGC-1 *α* expression. These results suggest that QSYQ increases myocardial fatty acid beta-oxidation and ATP content and improves myocardial energy metabolism and cardiac function most likely through interference in PPAR *α*/PGC-1 *α* pathway.

ATP 5D is the gene encoding ATP synthase subunit *δ* which contributes to the synthesis of ATP [13]. However, no study has been reported about the changes of ATP synthase subunits after DOX. The present study revealed that ATP 5D protein decreased significantly after DOX, probably accounting for the reduction of ATP content. Interestingly, treatment with QSYQ restrained the decrease of ATP 5D and the increase of AMP/ATP.

F-actins constitute myocardial thin filaments, which, alone with thick filament, are responsible for the actin-based myofilament motility [23]. We demonstrated in the present study the degradation of F-actin followed by the rupture of myocardial fibers and the hypofunction of cardiac contractility after DOX, an insult most likely due to the reduction of ATP content [24]. Furthermore, the beneficial role of QSYQ in maintaining the myocardial structure and cardiac function after DOX is presumably achieved through increasing the expression of ATP 5D and the synthesis of ATP leading to the preservation of F-actin.

Heart ejection function depends on cardiac energy supply and normal myocardial structure. We demonstrated that DOX caused cardiac energy depletion and myocardial structure damage, which contribute to the reduced heart ejection and perfusion function. QYSQ could restore the cardiac energy, myocardial structure and improve myocardial blood flow and cardiac function implying QYSQ as a promising remedy for reducing the adverse effects of DOX in clinic. 

In summary, QSYQ is able to ameliorate DOX-induced myocardial structure injury and cardiac dysfunction. This beneficial role of QSYQ is correlated with its potential to modulate energy metabolism, involving upregulating PPAR *α*/PGC-1 *α* and fatty acid oxidation, reducing myocardial FFA and increasing ATP level. This result suggests QSYQ as a potential management to cope with the obstacle that DOX confronts in clinical use, and provides insight for better understanding the mechanism behind the QSYQ effect. Nevertheless, the detailed mechanisms thereby QSYQ protects heart from injury by DOX need further clarification, and mor studies, particularly using lager animals, are required to verify the feasibility for QSYQ application in clinic. 

## Figures and Tables

**Table 1:** Echocardiography parameters of rat hearts in different conditions.

GROUP	*n*	LVIDd (mm)	LVIDs (mm)	LVPWd (mm)	LVPWs (mm)	EF (%)	FS (%)
Control 2W	6	7.468 ± 0.18	4.338 ± 0.14	1.985 ± 0.12	3.046 ± 0.06	71.03 ± 1.27	41.88 ± 1.07
DOX 2W	14	6.901 ± 0.023	4.985 ± 0.13*	1.372 ± 0.11*	1.780 ± 0.14*	51.68 ± 3.51*	27.49 ± 2.28*
Control 4W	4	7.654 ± 0.34	4.521 ± 0.21	1.873 ± 0.09	2.782 ± 0.07	69.73 ± 2.62	40.86 ± 2.27
DOX 4W + NS	6	7.624 ± 0.29	5.439 ± 0.29	1.395 ± 0.09^▲^	2.111 ± 0.32^▲^	53.52 ± 2.57^▲^	28.78 ± 1.64^▲^
DOX 4W + QSYQ	7	7.279 ± 0.10	4.106 ± 0.17^#^	1.565 ± 0.06^#^	2.428 ± 0.08^#^	72.93 ± 2.37^#^	43.60 ± 2.12^#^

*P < 0.05 versus Control 2W group;^ #^ *P* < 0.05 versus DOX 4W + NS group; ^▲^ *P* < 0.05 versus Control 4W group. LVIDd: left ventricular internal diameter at end-diastole; LVIDs: left ventricular internal diameters at end systole; LVPWd: left ventricular posterior wall at diastole; LVPWs: left ventricular posterior wall at systole; EF: left ventricle ejection fraction; FS: left ventricle fractional shortening. The data are presented as mean ± S.E. The treatments for each group are detailed in Section 2.
